# Tuberculin in Arthritis Deformans

**Published:** 1912-10-12

**Authors:** 


### TUBERCULIN IN ARTHRITIS DEFORMANS.

### Tuberculosis as an Ætiological Factor.

The ætiology of this disease is very obscure. The onset of the disease is frequently associated with some infective process, and Poincet and Leriche have laid great stress on the rôle played by tuberculosis. They maintain that fully 50 per cent. of cases are tuberculous in nature. The only means whereby definite information on the point can be obtained are the previous history or presence of a tuberculous lesion, the use of serodiagnostic methods, or animal inoculation with the arthritic products. The first two are not certain, and the third has seldom given positive proofs. All one can say at present is that, amongst the toxic processes that preside at the origin of chronic rheumatism, and especially progressive polyarthritis deformans, tuberculosis seems to hold an important place. It is impossible in most cases clinically to differentiate polyarthritis of tuberculous origin from those due to other causes.

Oppenheim and Lamy (*Le Progrès Medical*, July 27, 1912) give the result of their investigations on this point. By the use of Courmont's cutaneo-reaction with tuberculin they obtained a positive result in 54 cases of arthritis deformans out of 55, and they contrast this 98-per-cent. result with that obtained from a use of the test in aged hospital patients, in which case the percentage was 74. This would seem to indicate a greater frequency of present or antecedent tuberculosis in the case of arthritis, though the test is by no means an exact one. By the subcutaneous injection of tuberculin much more reliable information can be obtained. They thus investigated 49 cases of progressive polyarthritis deformans, 16 of whom were males and 33 females. These patients received two-tenths of a milligramme of purified tuberculin prepared at the Pasteur Institute, and where the cases did not react to this first injection a second of half a milligramme was given after eight hours, and where this also was negative, a third injection of 1 milligramme was given after a week. All the cases had a normal temperature of 37.5° before the test. As a result of the first injection, 8 were positive (the temperature rose one degree or more), 10 were doubtful (the increase in temperature was from four-tenths to nine-tenths of a degree), and 25 were negative (an increase of less than four-tenths of a degree or a diminution). After the second injection of these 25 negatives, along with 4 doubtfuls, 16 were positive, 7 were doubtful, and 6 were negative. These last 6 gave a very energetic reaction to the third injection. In a certain number of the cases—about 10—the febrile reaction was accompanied by pain in the affected joints. The obtaining of a positive tuberculin reaction is, however, not to be taken as a proof of the tuberculous nature of the arthritis. Some radiographers claim to be able to differentiate tuberculous arthritis deformans from that due to other causes by the appearances given in radiograms. Oppenheim is also of opinion that cases of tuberculous origin have generally a diminished arterial tension in contrast to the increased tension in other rheumatic cases. But, perhaps, the best proof that tuberculosis is an ætiological factor in the production of some would be the good effect of antituberculosis treatment. For more than a year Oppenheim and Lamy have had cases under tuberculin treatment.

They chose exclusively grave cases belonging to the classic type of progressive polyarthritis deformans in an advanced stage, in whom other treatments had proved ineffective. Six of their patients, for example, had unsuccessfully tried thyroid ex tract. The technique adopted was as follows: starting from the original tuberculin, as supplied by the Pasteur Institute, a series of dilutions of 1 in 100 were made (solution F), and from these several series of 1 in 1,000 (solution E), 1 in 10,000 (solution D), 1 in 100,000 (solution C), 1 in 1,000,000 (solution B), and 1 in 10,000,000 (solution A). With the last there was injected $\frac{1}{10}$ c.c., corresponding to $\frac{1}{1000000}$ mg. In the absence of febrile reaction the injections were repeated every five days, progressing each time by $\frac{1}{10}$, $\frac{2}{10}$, or $\frac{3}{10}$ c.c. Thus, when the injection was $\frac{9}{10}$ c.c. of solution A the following injection was $\frac{1}{10}$ c.c. of solution B, and so on. By slow and careful progress the doses were increased, so that at the end of treatment the patients were able to tolerate such elevated doses as 3 or 5 mg. tuberculin. Towards the end of treatment the interval between the injections was greater, 8 or 10 days elapsing between each.

Altogether 18 cases (4 men and 14 women) were so treated. Half of these were aged persons; the rest varied in age between 32 and 59 years. Six of these had pulmonary tuberculous lesions, 1 had a cold abscess, and 1 had an old coxalgia. The treatment was applied for six months to one year, with the result that 2 cases showed manifest amelioration, 4 showed slight but permanent amelioration, 2 showed slight but transitory improvement, and in 10 there was no good result. In none of them did the treatment produce any aggravation.

With regard to those classified as showing slight amelioration, this consisted specially in diminution or disappearance of pain. In 2 of these cases pain was abolished for several weeks but again appeared, whilst in 4 the absence of pain was maintained after the first injections. In 4 cases the mobility of the joints was increased, so that movements of which they were previously incapable became possible. This effect was only temporary in 1 case, and in the other 3 it was permanent. Another happy change was as regards the general health and strength. The appetite improved and the weight increased; in 2 this only lasted for some weeks; in 4 it was maintained. In all these cases a noticeable feature was the cessation of periodic exacerbations of temperature and pain.

Details are given of two advanced cases in which marked amelioration as a result of treatment was produced. The first case was a female patient, aged fifty-eight, who for six years had generalised arthritis deformans. The condition had started in the hands twenty-five years previously and the patient was absolutely impotent, incapable of any spontaneous movement of arms or legs, and the slightest attempt at passive movement provoked extreme suffering. The hands and wrists were typically deformed, the thighs and shoulders immobilised, the knees bent at a right angle, the ankles swollen, and the muscles atrophied. The patient was thin and cachectic. At the right apex there were signs of the presence of induration. The temperature was normal and the arterial tension low. She had to have five or six injections daily of ⅓ grain of morphia with a strong dose of sulphonal every night. Thyroid treatment produced no results. The tuberculin treatment, starting with very feeble doses, was continued for over a year. After the fourth injection, for the first time for several years, the nurses were able to move the patient without eliciting cries of pain. Slight spontaneous movement of the wrist became possible. The treatment had to be interrupted after two months, and there was a recrudescence of extreme pain which vanished again on the resumption of treatment. During the fourth month the morphia was much diminished, the general condition was considerably improved, and the appetite reappeared. The deformities, of course, were scarcely at all affected, but owing to the improvements in the wrist and elbow the patient was able to convey a spoon to her mouth—an action of which she had been incapable for many years. After a year of treatment her condition was considerably ameliorated, and though she was always impotent yet she could bear all necessary movements without suffering and could do without morphia almost completely.

The second patient, a woman of forty-nine, had generalised arthritis of six years' duration with typical deformity of hands, wrists, elbows, legs, and knees. Movement of the legs was very limited and walking was impossible. The general condition was good; there were no pulmonary lesions, no muscular atrophy, and the arterial tension was normal. From August 1911 till February 1912 she had thirty increasing injections of tuberculin without any appreciable modification of the condition being produced. After each injection there was an increase of articular pains without febrile reaction. In March amelioration began and went on increasing. The pains diminished and one could move the wrists, elbows, and knees fairly easily. In April, after the fortieth injection, the patient, who had been unable to leave her bed for five years, could now walk round the ward with the help of crutches.

In such extensively disorganised joints anything like cure is impossible, but there can be no doubt that the relief afforded to a patient whose life is an extreme of suffering is rather remarkable. The observations go a long way to prove the ætiological factor of tuberculosis in at least some cases of arthritis deformans.

